# Identifying the factors influencing the development of bilateral investment treaties with health safeguards: a Machine Learning-based link prediction approach

**DOI:** 10.1007/s42001-024-00341-z

**Published:** 2024-12-05

**Authors:** Haohui Lu, Anne Marie Thow, Dori Patay, Takwa Tissaoui, Nicholas Frank, Holly Rippin, Tien Dat Hoang, Fabio Gomes, Wolfgang Alschner, Shahadat Uddin

**Affiliations:** 1 Faculty of Engineering, The University of Sydney, 21 Ross Street, Forest Lodge, NSW 2037 Australia; 2 Leeder Centre for Health Policy, Economics and Data, The University of Sydney, 17 Johns Hopkins D, Camperdown, NSW 2050 Australia; 3 School of Regulation and Global Governance, Australian National University, 8 Fellows Rd, Acton, ACT 2601 Australia; 4 Special Initiative on NCDs and Innovation, WHO Regional Office for Europe, Marmorvej 51, 2100 Copenhagen, Denmark; 5 Faculty of Law, Monash University, 15 Ancora Imparo Way, Clayton, VIC 3168 Australia; 6 Pan American Health Organization/World Health Organization, Washington, DC USA; 7 Faculty of Law, University of Ottawa, 57 Louis-Pasteur Private, Ottawa, Ontario K1N 6N5 Canada

**Keywords:** Bilateral investment treaty, Feature importance, Machine learning, Network analysis, Link prediction

## Abstract

A network analysis approach, complemented by machine learning (ML) techniques, is applied to analyse the factors influencing Bilateral Investment Treaties (BITs) at the country level. Using the Electronic Database of Investment Treaties, BITs with health safeguards from 167 countries were charted, resulting in 534 connections with countries as nodes and their BITs as edges. Network analysis found that, on average, a country established BITs with six other nations. Additionally, we used node embedding techniques to generate features from the network, such as the Jaccard coefficient, resource allocation, and Adamic Adar for downstream link prediction. This study employed five tree-based ML models to predict future BIT formations with health inclusion. The eXtreme Gradient Boosting model proved to be superior, achieving a 64.02% accuracy rate. Notably, the Common Neighbor centrality feature and the Capital Account Balance Ratio emerged as influential factors in creating new BITs with health inclusions. Beyond economic considerations, our study highlighted a vital intersection: the nexus between BITs, economic growth, and public health policies. In essence, this research underscores the importance of safeguarding public health in BITs and showcases the potential of ML in understanding the intricacies of international treaties.

## Introduction

Bilateral Investment Treaties (BITs) are a subtype of International Investment Agreements (IIAs) enacted by governments to stimulate foreign investment as part of broader economic policy objectives. The intent of these treaties is to attract foreign capital, foster economic growth, and promote inter-country cooperation [1]. While intended to foster mutually beneficial environments, these treaties have also sparked challenges, especially over public health issues such as tobacco control, and climate change. In these circumstances, governments often find themselves in a precarious position, struggling to balance investor demands with their obligation to protect public health, thus highlighting the complex legal and ethical dilemmas in treaty design and implementation [2].

Over the past six decades, BITs have emerged as the premier international legal tool for promoting and overseeing foreign direct investment [1]. The peak in BITs creation occurred from the mid-1980s to the mid-2000s, marking the zenith of their expansion [1]. These treaties between governments usually provide extensive rights to foreign investors. This includes safeguarding contractual rights and the privilege to sue governments and seek international arbitration should any disagreements over investments arise [1]. Further, BITs have inadvertently enabled some businesses (the investors) to challenge and influence health-related regulations [3]. Recognising these risks, health experts advocate for the integration of health protection clauses directly into these agreements [4]. However, implementing such provisions has been inconsistent and gradual, reflecting a lack of engagement from and with the health sector and insufficient knowledge about optimal protective clauses to incorporate [5]. Over time, remarkable uniformity in the composition of health safeguard clauses has been observed [2].

The novelty of our study lies in its comprehensive analysis of the factors that enable the inclusion of robust health safeguards in IIAs, using machien learning (ML) methods. Identifying the factors enabling the inclusion of robust health safeguards in IIAs can provide insights into global economic governance and trade dynamics, therefore aiding in tracking the diffusion of diverse policy concepts, norms, and standards globally [6]. Evaluating country-level measures, such as foreign direct investment, Gross Domestic Product (GDP), and population, can provide insights into patterns in the incorporation of health inclusion in BITs. Our research is motivated by the need to ensure that international economic collaborations do not compromise public health, and to provide actionable strategies for public health actors to advocate for stronger health protections in future treaties.

Our primary research questions are: What are the characteristics associated with the inclusion of robust health safeguards in BITs? How can public health advocates effectively promote the inclusion of these safeguards in upcoming IIAs? To address these questions, we employ a novel approach that combines network analysis techniques with machine learning methods to derive insights that can improve decision-making regarding the integration of health safeguards within IIAs.

The rationale for using a network analysis is that it allows us to capture the intricate relationships and dependencies between various BITs and their health-related provisions. This method is particularly well-suited for analysing complex networks, such as those formed by international treaties, where the connectivity and centrality of nodes (treaties) can significantly impact the diffusion of health safeguards. The methodology involves several key steps:

- Compiling a comprehensive dataset to construct a network map of existing BITs that have incorporated health-related provisions.
- Generating network features, such as degree centrality, to gain a deeper understanding of the network’s topology. This involves analysing how these BITs are linked to each other and the extent to which certain treaties are central or peripheral in the context of health-related provisions.
- Implementing a link prediction task within the IIA network. This task aims to identify factors, such as GDP and population size, that are associated with the likelihood of including health-related provisions in IIAs. We can anticipate which countries or treaties are more likely to adopt health safeguards in the future by predicting potential links.

We aim to deepen our understanding of how health safeguards are integrated within IIAs. The insights gained will serve as a foundation for further research and the development of policy recommendations. These recommendations are intended to ensure that international economic collaborations do not compromise the health and well-being of global communities, maintaining a harmonious balance between economic progress and public health preservation.

## Related work

**Health safeguards in bilateral investment treaties (BITs)**: The integration of health safeguards in BITs has been a growing area of interest among researchers and policymakers. Uddin et al. [7] highlights the complex interplay between investor protections and public health obligations, demonstrating that while BITs aim to promote economic growth, they can also pose significant challenges to health regulation. McNeill et al. [4] argue for the necessity of embedding health protection clauses within these treaties to mitigate adverse impacts on public health. However, Bonnitcha et al. [5] point out the inconsistency and gradual implementation of such provisions, attributing this to a lack of engagement from the health sector and insufficient knowledge about effective health safeguards. These studies underline the critical need for a systematic approach to incorporating health safeguards into BITs, ensuring that public health is not compromised by international investment agreements.

**Machine learning in BITs**: Machine learning has begun to play a crucial role in the analysis and prediction of patterns within BITs. For instance, Alschner and Skougarevskiy [6] utilised text-as-data approaches and machine learning techniques to map and analyse the universe of international investment agreements, highlighting how such methodologies can reveal the diffusion of treaty norms and standards over time. Moreover, studies demonstrated the potential of machine learning in predicting the content of future international law and BITs based on existing agreements, providing a data-driven foundation for understanding treaty evolution [8, 9]. These studies illustrate that machine learning is not only instrumental in dissecting the complex structure of BITs but also in anticipating changes and trends within this legal framework.

**Feature importance in forming BITs with health inclusion**: Understanding the key factors that influence the inclusion of health safeguards in BITs is essential for developing effective policies. Feature importance analysis helps identify the most significant attributes that drive the adoption of health-related clauses. Researches have highlighted how investor protections can conflict with public health regulations, suggesting that economic indicators such as GDP, foreign direct investment levels, and population size play crucial roles in this dynamic [10–12]. Researchers can uncover which factors are most predictive of health safeguard inclusion, providing a data-driven foundation for advocating more robust health protections in future BITs using machine learning technique. This approach not only enhances our understanding of the determinants of health-inclusive treaties but also aids in the formulation of targeted strategies to promote public health within the framework of BITs.

## Materials and methods

### Dataset

The BIT data for this study was obtained from the Electronic Database of Investment Treaties (EDIT) [13]. The dataset is available at: https://edit.wti.org/document/investment-treaty/search. This extensive database includes a total of 3,298 BITs (status: December 2021), which cover a wide range of statuses, including active, pending, and even terminated treaties. This study extracted meta-information from all BITs mentioning ‘health’ along with the adjacent article text. Based on prior analysis by the EDIT, 18% (584 out of 3298) of BITs have 934 unique mentions of health [2]. We omitted 27 unclassified BITs (without any classification in Thow et al. [2]) and two BITs that involved multiple countries or regions from the 584 BITs under consideration for analysis. As previous studies have indicated, the likelihood of BIT creation between countries is greater when they are larger and similar in GDP, geographically closer, either adjacent or non-adjacent with a shared language, have higher political stability and lower expropriation risks, and possess a more skilled labour force or a broader capital/unskilled labour ratio [14]. In addition to the BIT data, our study meticulously incorporated a comprehensive array of country-level economic and social indicators relevant to the year of each agreement. These indicators encompass the current account balance, foreign direct investment, GDP, Human Development Index (HDI), inflation, life expectancy at birth, literacy rate, population, trade (as a percentage of GDP), and unemployment. Crucially, for each country involved in the BITs, these indicators were sourced from the World Bank Databank [15] corresponding to the specific year when each BIT was signed. This approach ensures the provision of reliable and temporally accurate economic and social statistics, offering a nuanced understanding of the context surrounding each agreement within the span of our 50-year study period.

### Bilateral investment treaties network

Network analytics involves examining intricate networks to understand their structure, dynamics, and evolution. This field investigates the relationships and trends observed within a network, typically represented as a graph comprising nodes and connecting lines, known as edges. In our research, we use a BIT network to describe the relationship between BITs. The representation is as follows:

1 $$\begin{aligned} G=(N,E) \end{aligned}$$

**Fig. 1:**
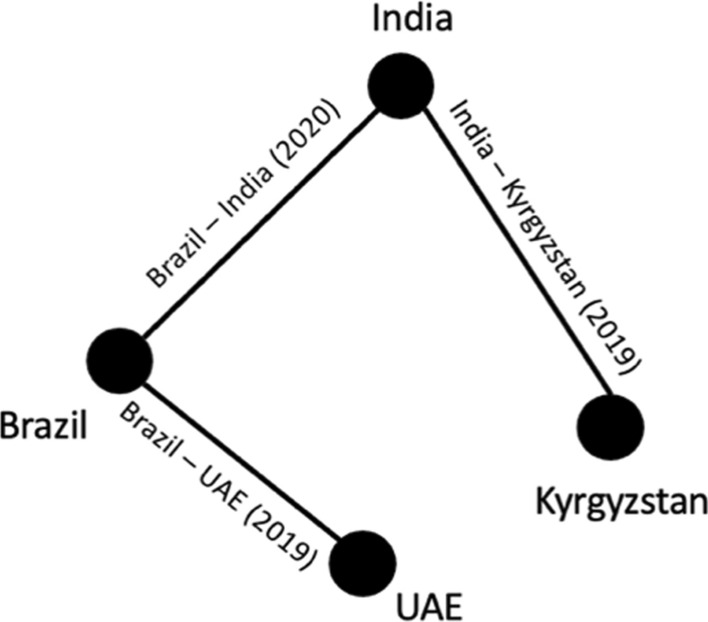
Example of a BIT network

Where *G* denotes the BIT network from the given dataset, and *N* represents nodes, each corresponding to a different country. An edge *E* signifies the presence of a BIT between two countries, connecting them. Figure 1 provides an example of a BIT network among four countries: Brazil, India, UAE, and Kyrgyzstan. Three BITs are depicted: between Brazil and India in 2020, Brazil and UAE in 2019, and India and Kyrgyzstan in 2019.

#### Link prediction

**Fig. 2:**
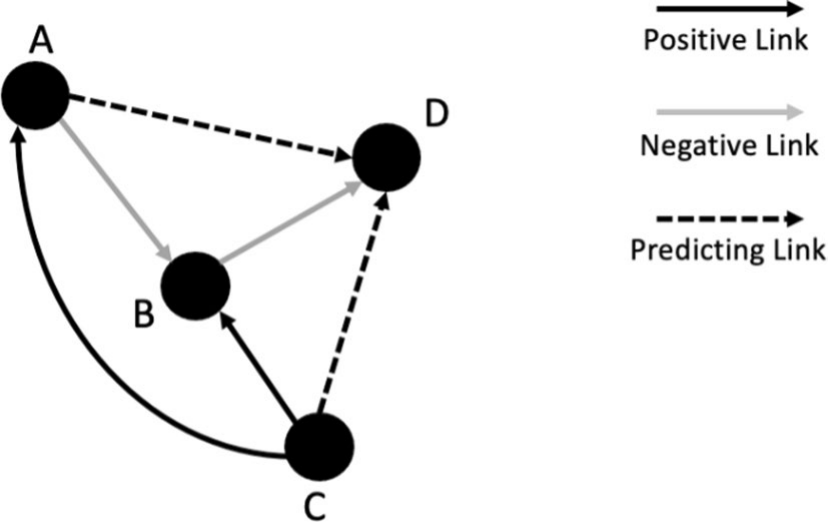
Link prediction. The figure shows a graph with nodes A, B, C, and D, connected by arrows indicating positive links (solid lines), negative links (grey lines), and predicting links (dashed lines)

Link prediction entails forecasting the probability of a connection occurring between two nodes within a network. This process is valuable in anticipating upcoming connections, identifying omitted links, and signalling potential interconnections which may be significant for the integrity and evolution of the network. In Fig. 2, four nodes are represented, labelled A through D. Positive existing connections are illustrated between nodes C and B, C and A, indicating active relationships. Conversely, negative links are depicted between nodes A and B, and B and D, suggesting an absence of a relationship. The figure further ventures into the realm of predictive analytics by proposing potential future links. It suggests a likelihood of a connection forming between node A and D, as well as between C and D. Such predictions are informed by the network’s current structure and the dynamics of node interactions, using complex algorithms that take into account various factors such as the network’s topology, node similarity, and existing connection patterns.

Several methods for link prediction include:

- Common Neighbours: The number of common neighbours shared by two nodes is used to predict links [16]. Besides using Common Neighbours, our research also incorporates the concept of common neighbour centrality. This method is grounded in two fundamental characteristics of nodes: the count of mutual neighbours they share and their centrality values within the network. Here, a mutual neighbour means nodes shared between two distinct nodes, while centrality indicates the significance or prominence of a node within the network [17]. The Common Neighbours link prediction algorithm posits that the more common neighbours two nodes have, the more likely they are to form a link. In Fig. 2, nodes A and B, with two common neighbours (C and D), would have the highest likelihood of forming a link. The node pair CD, with no common neighbours, would be the least likely to form a link according to this algorithm.
- Jaccard Coefficient: The similarity of two nodes’ neighbour sets is calculated by dividing the number of common neighbours by the total number of unique neighbours [18]. For nodes A and C in Fig. 2, which share one common neighbour (D) and have three unique neighbours between them (B, C, and D), the Jaccard Coefficient would be 1/3.
- Preferential Attachment: This assumes that higher-degree nodes are more likely to make new connections [19]. For nodes A and C, with a common neighbour D, if D has a degree of 2, the Preferential Attachment score would be 1/log(2).
- Adamic Adar: It measures the similarity between two nodes based on their common neighbours, giving more weight to common neighbours with low degrees [20]. The formula for the Adamic Adar index between nodes A and D would be described as: sum up one divided by the log of the number of connections for each node that is directly connected to both A and D. Thus, the Adamic Adar index for nodes A and D is 0.9102
- Resources Allocation: It considers common neighbours but assigns weights based on their degree and calculates the similarity score as the sum of the resources allocated to their common neighbours [21]. The score is calculated similarly to Adamic-Adar but without taking the inverse logarithm. For nodes A and D, it sums the reciprocal of the number of connections for each node that is a common neighbour to both A and D. The Resource Allocation index for nodes A and D is 1/3.

For the Common Neighbour, Jaccard Coefficient, Preferential Attachment, Adamic-Adar and Resource Allocation, the higher the calculated score, the higher the predicted likelihood of a link forming between two nodes. These scores are often used to rank potential links in order of their likelihood of forming, with higher scores indicating a higher priority or probability. However, existing similarity-based methods focus solely on the network structure, overlooking the node’s individual features.

#### Node embedding

Node embeddings offer a sophisticated approach to link prediction in networks by combining node-specific features with traditional structural indices. The aim of this study is to perform link prediction in the BIT network, identifying critical features that influence the establishment of new BIT. In such network, where nodes represent countries, it’s crucial to include country-specific features, such as GDP and population, to fully understand network dynamics. Our approach integrates these structural indices with key economic indicators to create comprehensive node embeddings. This fusion results in vector representations that encapsulate both network structure and node features, providing a rich dataset for link prediction. Our link prediction model benefits from a multifaceted view of network connections by leveraging node embeddings, significantly enhancing predictive accuracy. This method transcends traditional link prediction by incorporating economic, demographic, and other relevant country-specific factors, offering a more complete picture of potential link formations in BIT networks. In addition, the country-specific features will be used as a ratio for the task of link prediction. As an illustration, the formula for the GDP ratio between two countries with a BIT is

2 $$\begin{aligned} \text {GDP ratio} = \frac{\text {Lower GDP between two countries}}{\text {Higher GDP between two countries}} \end{aligned}$$

Using ratios instead of differences between two countries offers several benefits: it normalises the feature, and the range falls between 0 and 1, which is advantageous for machine learning techniques.

#### Creation of training and testing dataset

For link prediction through supervised learning, the creation of separate training and testing datasets is essential. Our approach involves designing subgraphs that maintain a consistent number of nodes but vary in the number of edges to appropriately train and evaluate ML models. We begin by selecting an equal number of non-edges, which represent potential but currently non-existent links in the BIT network, as negative samples. Subsequently, incorporating these negative samples, we partition the data in an 80:20 ratio. In this case, 80% is dedicated to training ML models, while the remaining 20% serves for testing purposes.

#### Period analysis of BIT networks

To cross-validate the network dynamics of BIT formation, we segmented the data into three distinct periods: 1959–1985, 1986–2005, and 2005–2021. These divisions are based on historical trends in BIT creation. Specifically, the period from 1986 to 2005 [1], marks the peak of BIT establishment and represents a significant expansion phase. This peak period was therefore selected for detailed analysis, allowing us to closely examine the height of BIT activity. The periods before and after this peak offer valuable contrasts, depicting the initial establishment and subsequent consolidation of BIT networks, respectively.

### Link prediction models

#### Machine learning method

ML methods emulate human intelligence by discerning patterns from available data. For our link prediction task, we employ a supervised ML approach. One of the primary methods we use is a decision tree, a flowchart-like structure in which each internal node represents an attribute, each branch represents a decision rule, and each leaf node represents an outcome. Decision trees offer intuitive visualisations and can handle categorical and numerical data [22]. In addition, we utilise Random Forests, which enhance decision-making by using multiple decision trees, thereby introducing diversity and reducing the correlation between them [23]. Furthermore, we incorporate eXtreme Gradient Boosting (XGBoost), a gradient-boosting library renowned for its efficiency, parallel processing and handling of missing values. This results in improved model performance and shorter training times [24]. In addition, we also employ AdaBoost, which combines the outputs of multiple weak classifiers to create a strong classifier, thus improving the robustness of the predictions [25]. Additionally, we use CatBoost, a gradient-boosting algorithm that is particularly effective with categorical features and reduces overfitting while providing high performance [26].

#### Evaluating classifiers

This research calculated the confusion matrix, which summarises the effectiveness of a classifier as evaluated on a test dataset. This matrix is commonly employed for computing various performance metrics, such as accuracy, recall, precision, and F1 score. The performance metrics were derived from the confusion matrix using the formulas provided below:

3 $$\begin{aligned} \text {Accuracy}= & \frac{TP + TN}{TP + TN + FP + FN} \end{aligned}$$

4 $$\begin{aligned} F_1 \text { score}= & \frac{2 \times TP}{2 \times TP + FN + FP} \end{aligned}$$

5 $$\begin{aligned} \text {Precision}= & \frac{TP}{TP + FP} \end{aligned}$$

6 $$\begin{aligned} \text {Recall}= & \frac{TP}{TP + FN} \end{aligned}$$

### Feature selection technique

The feature selection technique applied in this research involves leveraging the feature importance scores from various machine learning models. These importance scores are calculated using specific methods inherent to each model. The models used include XGBoost, CatBoost, Random Forest, AdaBoost, and Decision Tree, each providing a mechanism to evaluate and rank the significance of each feature. In this section, details of feature selection for each model are provided.

#### Decision tree and random forest

For Decision Tree and Random Forest, the feature importance is calculated based on the mean decrease in impurity (Gini importance) [22, 23]. When a feature is used to split a node, the impurity of the node is decreased. The importance of a feature is computed as the total reduction of the criterion (impurity) brought by that feature across all trees in the forest. The feature importance for each feature $j$ is calculated as:

$$\begin{aligned} I_j = \sum _{t=1}^{T} \Delta i_t \cdot \frac{n_t}{N} \end{aligned}$$

Where $\Delta i_t$ is the decrease in impurity from splitting on feature $j$ at node $t$. $n_t$ is the number of samples reaching node $t$. $N$ is the total number of samples. $T$ is the total number of nodes in the tree.

#### AdaBoost

In AdaBoost, feature importance is computed based on the weight of each weak learner and the feature’s contribution to reducing the classification error. The importance of a feature is the sum of the importance of the feature in each tree, weighted by the weight of that tree. For each feature $j$, the importance $I_j$ is the sum of the importances of the feature in each weak learner $m$, weighted by the weight $\alpha _m$ of that learner:

$$\begin{aligned} I_j = \sum _{m=1}^{M} \alpha _m \cdot I_{j,m} \end{aligned}$$

Where $\alpha _m$ is the weight of the $m$-th weak learner. $I_{j,m}$ is the importance of feature $j$ in the $m$-th weak learner, $M$ is the total number of weak learners [25].

### Summary of the proposed method

**Fig. 3:**
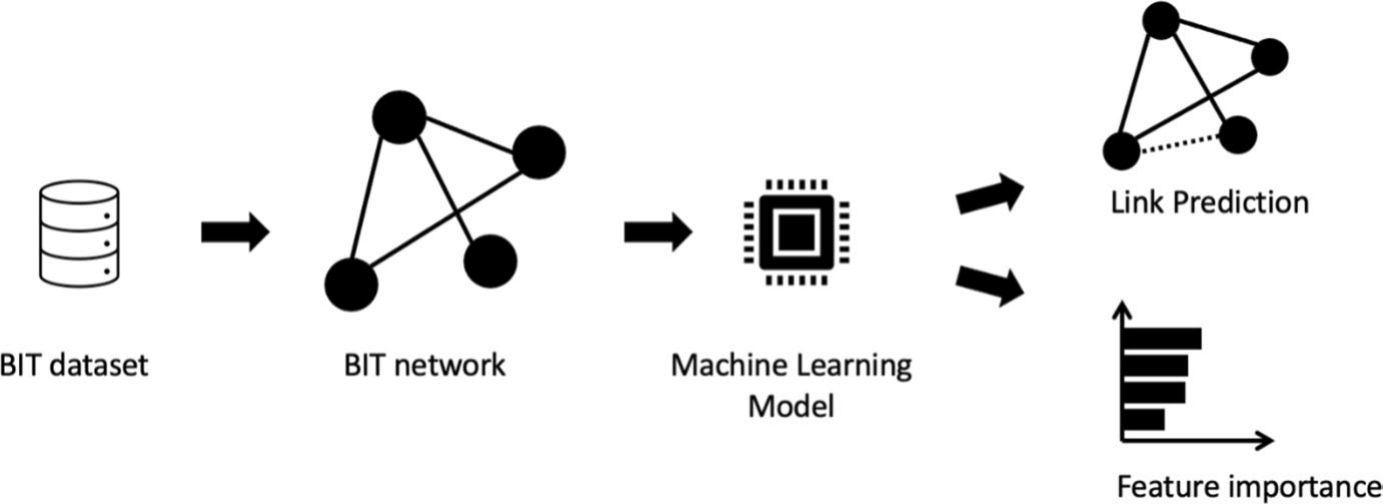
Summary of the proposed method

Figure 3 illustrates the link prediction task. EDIT data is utilised as input to construct a network, where countries are nodes, and an edge signifies a BIT between two countries. The input to our models consists of data from countries involved in BITs, including features such as capital account balance, unemployment rate, and other socio-economic indicators. The output of the models is a binary classification indicating whether a link (i.e., a BIT) exists between the countries. This BIT network is then analysed using machine learning techniques to predict potential future BIT connections, a process known as link prediction. The periods chosen for analysing the network dynamics of BIT formation-1959–1985, 1986–2005, and 2005–2021-are grounded in historical trends in BIT creation. The second period, 1986–2005, represents the peak of BIT establishment [1], marking a significant phase of expansion. This peak justifies its selection as a distinct period for focused analysis, allowing us to explore the zenith of BIT activity. The preceding and subsequent periods provide contextual contrasts, capturing the initiation and consolidation phases of BIT networks, respectively. In this context, link prediction aims to forecast which pairs of countries without a current treaty are likely to establish one, based on a variety of factors such as economic conditions, historical treaty data, and the specific time period. This problem is treated as a binary classification task, where potential links are categorised as positive (likely to result in a BIT with health inclusion) or negative (unlikely to do so). This research seeks to identify key factors that influence the formation of BITs by assessing the importance of different node features in this classification, including temporal trends. This approach not only predicts future BIT formations but also sheds light on the underlying drivers of these BITs. While current state-of-the-art models like Graph Neural Networks (GNNs) offer powerful performance for such tasks, they are often considered black-box models and lack interpretability [27]. Given that our audience is not primarily from the computer science or machine learning fields, we prioritise the use of tree-based models due to their explainability and ease of interpretation [28].

## Results

This section outlines the BIT network’s topology, the efficiency of ML algorithms across different time periods (1959–1985, 1986– 2005, and 2005–2021) for various types of BITs (defensive, neutral, and progressive), and the top-performing model’s essential features, emphasizing the temporal dynamics and strategic nature of BIT formation.

### Network topology

We generated the BIT network using the method described in Sect. 2.2. Grasping a network’s structure and the functions of its nodes is a fundamental aspect of network analysis. Table 1 presents the measurements based on social network principles.

**Table 1:** Summary of network properties

Network properties	Value
Number of nodes	167
Number of edges	534
Average degree	6.3952
Average path length	2.5811
Average clustering coefficient	0.1390
Average shortest path	0.5706

**Fig. 4:**
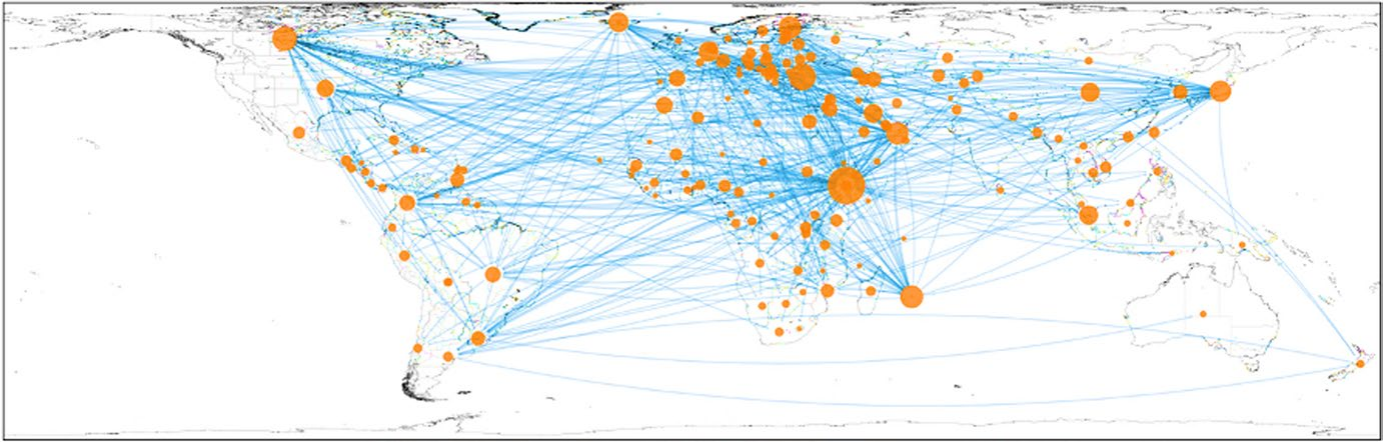
The visualisation of the generated network of BITs with health inclusions. Nodes are the countries. The edges refer to the link between countries that have BITs with health provisions

The BIT network analysed includes 167 countries and 534 connections, focusing solely on BITs that incorporate health provisions. This approach not only highlights the unique characteristics of countries that negotiate health-inclusive BITs but also offers nuanced insights into the strategic importance of health in these agreements. We delve into the specific factors that influence the inclusion of health in international treaties by examining BITs with health provisions, providing a richer understanding of the underlying dynamics at play. The number of edges (534) is slightly lower than the total count of BITs. This discrepancy is due to some countries having established multiple BITs with the same partner countries. On average, each country has BITs with approximately six other countries, as indicated by an average degree of 6.3952. The average path length in the BIT network is 2.5811, signifying that it takes about 2.58 steps on average to connect any two countries through their BIT relationships. The network’s density is approximately 0.139, implying relative sparsity when considering all possible BIT connections. The average clustering coefficient is around 57.06%, suggesting a significant level of interconnection among countries, indicating that countries with BITs with a common partner also tend to have BITs with each other. Figure 4 provides a detailed visual representation of the BIT network, which is exclusively composed of treaties that incorporate health provisions, underscoring the dataset’s specific focus. This illustration captures the extensive and interconnected nature of these health-inclusive BITs, showcasing the complex relationships within the global network. Notably, the treaties with health inclusions span parties from all major regions of the world, highlighting the widespread acknowledgment of health as a critical component in international agreements. This visualisation not only clarifies the scope of our analysis-restricted solely to BITs with health provisions-but also begins to elucidate the varied implications of such inclusions across different geopolitical landscapes. We can delve deeper into understanding how health considerations are integrated into BITs by concentrating on this dataset, reflecting the strategic priorities of countries and regions in promoting health within the framework of international trade and investment.

### Performance of machine learning models for link prediction

We utilised three tree-based machine learning models for link prediction: decision tree, random forest and XGBoost. Before modelling the training data, we produced an equal amount of negative samples matching the number of edges in the network, resulting in a dataset comprising 534 positive and 534 negative edges. Subsequently, the dataset was partitioned into an 80-20 split for training and testing. Hyperparameter tuning was conducted for these models using the Sklearn package [29] in Python.

**Table 2:** Comparison of machine learning models’ performance

model	Accuracy (%)	Precision (%)	Recall (%)	F1-score (%)
Decision tree	55.14	55.25	55.24	55.12
Random forest	60.28	60.23	60.21	60.21
AdaBoost	56.54	56.54	56.55	56.53
CatBoost	62.15	62.15	62.16	62.14
XGBoost	64.02	64.05	64.06	64.02

Table 2 presents a comparison of the performance of five machine learning models across various metrics. The Decision Tree model achieved an accuracy of 55.14% and an F1-score of 55.12%. The Random Forest model showed better performance with an accuracy of 60.28% and an F1-score of 60.21%. CatBoost performed even better, with an accuracy of 62.15%, precision of 62.15%, recall of 62.16%, and an F1-score of 62.14%. The AdaBoost model demonstrated slightly lower results, with an accuracy of 56.54%, precision of 56.54%, recall of 56.55%, and an F1-score of 56.53%. In contrast, XGBoost outperformed all other models, achieving an accuracy of 64.02%, precision of 64.05%, recall of 64.06%, and an F1-score of 64.02%. Overall, XGBoost was the most effective model for this dataset, demonstrating state-of-the-art performance for gradient boosting techniques.

### Feature importance in the best-performed model

**Fig. 5:**
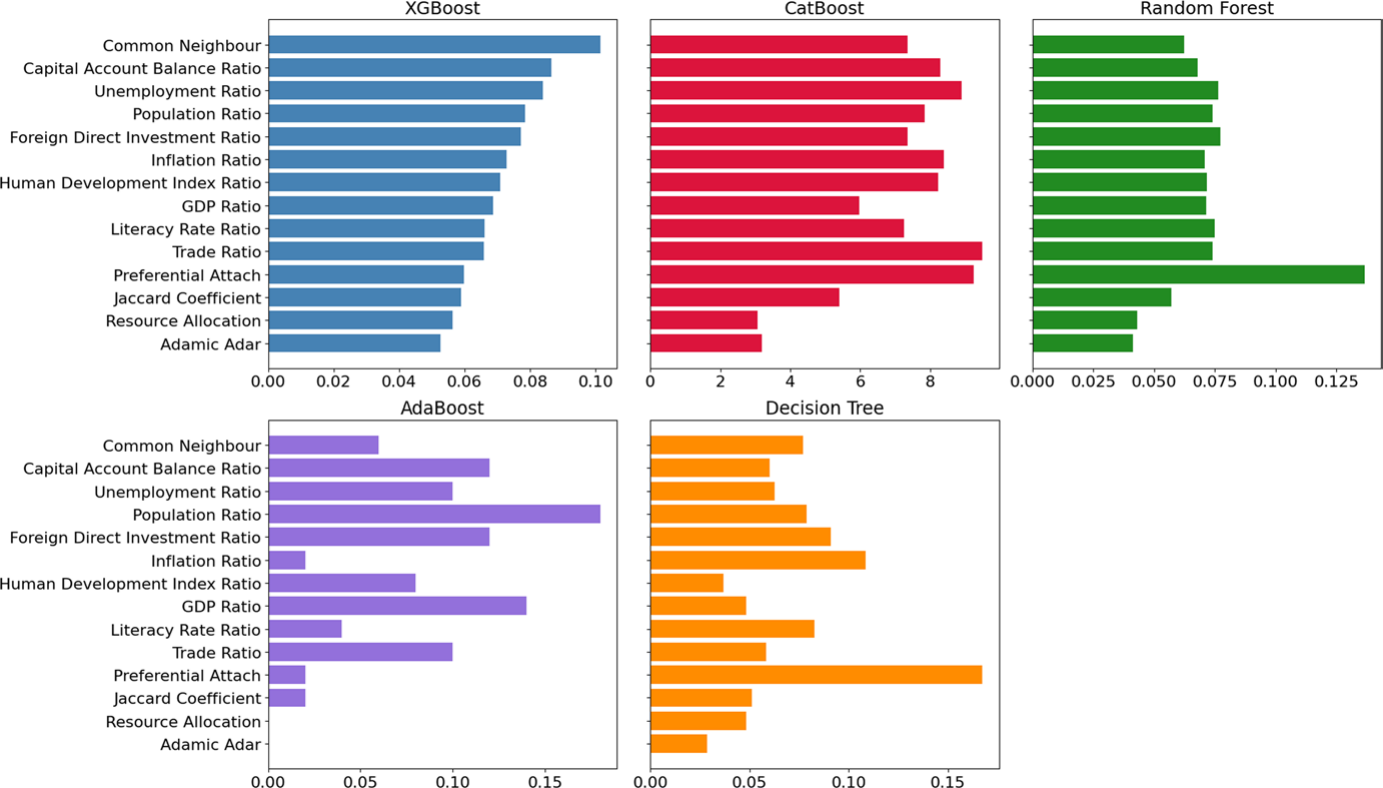
Feature importance for machine learning models

Figure 5 presents a picture of how the importance values of various factors, identified through the ML models, influence the likelihood of countries forming BITs with health provisions. Possible characteristics to include were selected based on the literature [14] and were finalised during a brainstorming exercise of the authors and the broader project team, including Jaccard Coefficient, Resource Allocation, Adamic Adar, Preferential Attach, Common Neighbour, Capital Account Balance Ratio, Foreign Direct Investment Ratio, GDP Ratio, Human Development Index Ratio, Inflation Ratio, Literacy Rate Ratio, Population Ratio, Trade Ratio, and Unemployment Ratio.

Common Neighbour is highly valued across all models, suggesting that countries with shared connections are more inclined to establish BITs with each other, notably when they include health safeguards. This is also in line with the conclusion from Mark S Manger, et al. [30] et al.: Countries are more likely to enter into BITs with nations to which they already have indirect connections. Capital Account Balance Ratio and Unemployment Ratio also frequently rank high, reflecting the models’ emphasis on economic stability and employment levels as critical predictors.

However, there are notable differences in how each model assigns importance to specific features. For instance, XGBoost and CatBoost give considerable weight to features like Foreign Direct Investment (FDI) Ratio and Population Ratio, indicating a nuanced consideration of international investments and demographic factors. Conversely, models like Random Forest and Decision Tree may show more variation in feature importance, potentially due to their inherent algorithmic differences, such as Random Forest’s reliance on multiple decision trees that can capture more diverse patterns within the data. Additionally, AdaBoost displays a different pattern, with feature importance scores more evenly distributed, reflecting its iterative boosting approach, which adjusts weights to focus on harder-to-predict instances. Despite these differences, the models are very similar in their overall approach to feature importance, emphasising key economic and demographic factors in their predictions.

Understanding these dynamics is critical as it sheds light on the strategic considerations nations undertake when negotiating BITs, particularly those that also prioritise health. It showcases that beyond mere economic agreements, these treaties are strategic tools used by countries to align with partners that share similar economic profiles or network positions, aiming to address common health challenges through international cooperation.

### Temporal insights and variations in BIT types

**Fig. 6:**
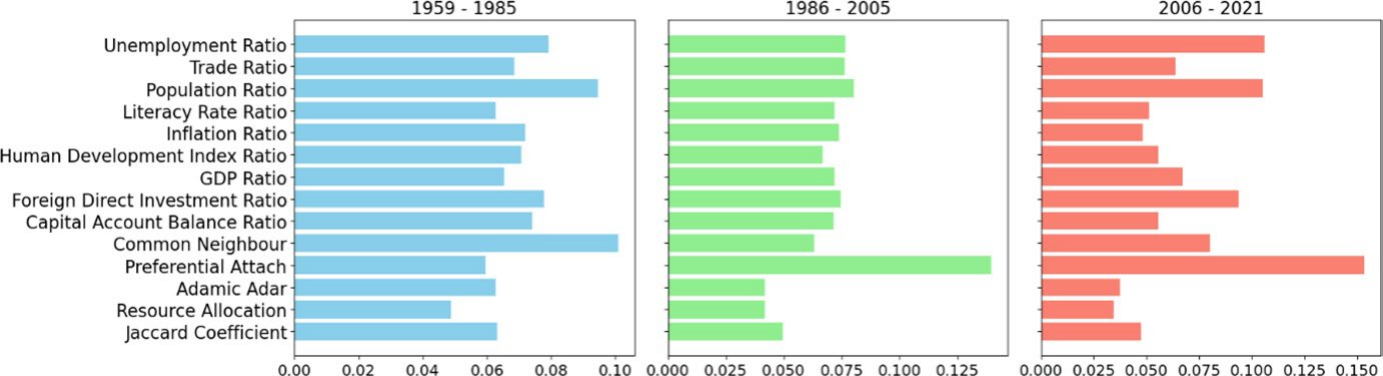
Feature importance for different periods

Given the literature indicates that the zenith of BIT creation spanned from the mid-1980s to the mid-2000s [1], we partitioned the BIT network into three distinct sub-networks, based on the signature dates of BITs: from 1959 to 1985, between 1986 and 2005, and from 2006 to 2021. Figure 6 shows the change in feature importance over three distinct periods: 1959–1985, 1985–2005, and 2006–2021. In the initial period (1959-1985), we observe that the Common Neighbour, which are typically associated with network connectivity and link prediction in graph theory, appear to be the most significant features. Moving to the period 1985–2005, there is a noticeable shift towards preferential attachment, which means that the more connected a country is, the more likely it is to receive new BITs. This underscores the path dependency effect-once a country commits to a specific investment pathway, it tends to adhere to it. Reasons for this may include efficiency gains from using established text, bureaucratic inertia, personal investment by bureaucrats, and the cost of deviation. In the most recent period (2006–2021), preferential attachment remains one of the most important features, with trends in economic indicators such as the unemployment rate and population ratio becoming increasingly influential. This evolution suggests a transition from network-based features to more traditional economic indicators over time, hinting at changes in the underlying factors driving the outcomes of interest. The analysis of these periods reveals how the importance of various features has shifted, possibly reflecting the changing dynamics of the global economy and policy focus.

**Fig. 7:**
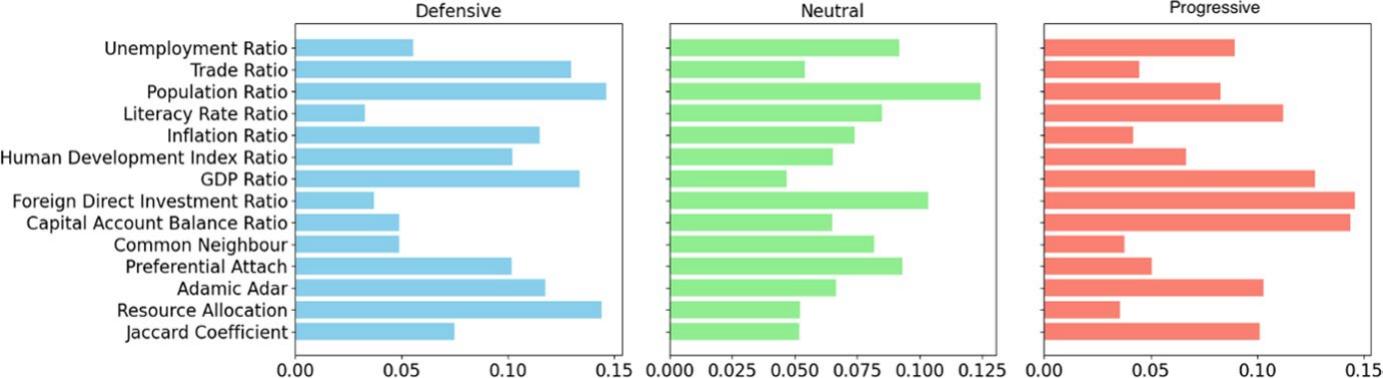
Feature importance for various type of BITs

Figure 7 illustrates the feature importance for various types of health clauses in BITs, namely Defensive, Neutral, and Progressive [2], revealing a common emphasis on network and economic indicators across all categories. Defensive BITs emphasise protective features such as the Resource Allocation, and population ratio, while Neutral BITs concentrate on country level measures like the population ratio and foreign direct investment ratio, and Progressive BITs give priority to country level measures such as foreign direct investment ratio and capital account balance. These differences highlight the diverse strategic approaches that countries adopt in BIT negotiations, reflecting their unique economic and diplomatic objectives.

## Discussion

BITs have become instrumental in attracting foreign investment and spurring economic growth. However, their influence extends beyond economics, sometimes interfacing with public health policies [31]. Industries have used them to challenge health regulations, especially in tobacco control and medicine access [32, 33]. This tension has sparked debates among policymakers and health experts. While governments use these tools to boost foreign investment, there is a growing concern about the potential undermining of health policies. Recognising these risks, the WHO has advocated for health safeguards within IIAs, but the adoption of and reference to WHO regulations in IIAs remains inconsistent, pointing to a need for greater awareness and engagement.

In this study, we conducted a network analysis of BITs with health inclusions. Within this BIT network, we observed that BITs with health inclusions foster complex international relations, with the average country in our dataset having treaties containing health inclusions with about six other nations. This interconnectedness is further highlighted by the average clustering coefficient, which suggests countries often engage in BITs with nations that share common partners. The BIT literature has traditionally relied on regression to model BIT determinants [34, 35]. This approach is superior precisely because it avoids relying exclusively on monadic or dyadic attributes, but rather takes into account network dynamics and processes. Further, another finding of the study is the substantial variation in health protection provisions within BITs. Although health experts have advocated for robust health protection measures, their implementation has been inconsistent and sporadic. This discrepancy might stem from the dominant influence of economic interests or a lack of awareness among negotiators regarding the public health implications of these treaties [5]. This study reveals fluctuations in health protection within BITs, suggesting economic interests or negotiator awareness could influence their inconsistent inclusion. An analysis of the BIT network over time (i.e., pre mid-1980s, mid-1980s to the mid-2000s, and after mid-2000s) shows an early and middle phase emphasis on network features, and a recent shift towards economic indicators, reflecting changes in global economic priorities which may affect health protection provisions in BITs.

Using machine learning models, our study effectively identified factors that influence the inclusion of health safeguards in BITs. Overall, among the three machine learning models, XGBoost performed the best. The "Common Neighbour" network feature from the BIT network with health inclusion emerged as the most crucial feature. This suggests that mutual connections between countries, possibly influenced by geographical, historical, or economic ties, play a significant role in the formation of BITs with health safeguards. Additionally, the prominence of features such as the Capital account balance ratio and Unemployment rate Ratio emphasises that economic and developmental contexts are crucial when countries negotiate BITs with health inclusion. This could suggest that nations prefer to establish BITs that include health provisions with other countries that share comparable economic or developmental paths. In addition to our quantitative findings, we provide qualitative insights that illustrate the practical impact of our model’s predictions. For instance, visualisations of the BIT network highlight how countries with strong mutual connections tend to include more robust health safeguards. On the other hand, BITs between countries with shared economic and developmental contexts have led to enhanced public health initiatives, such as improved access to essential medicines and stronger tobacco control measures. These insights underscore the importance of economic and network factors in shaping BITs with health provisions, providing a clearer understanding of the conditions that favour their inclusion.

In light of our findings that connections with countries and economic alignment influence the inclusion of health safeguards in BITs, future research could explore strategies for leveraging these insights to strengthen health provisions. The findings can be applied in policy formulation, public health advocacy, and economic diplomacy, solving problems related to balancing economic growth with public health priorities, and can be used by policymakers, public health organisations, development agencies, researchers, and legal experts. For instance, policymakers might use this knowledge to identify potential BIT partners with shared health objectives or similar economic development levels, aiming to foster agreements that better address public health concerns. Additionally, this understanding could guide capacity-building efforts in negotiations, ensuring that health inclusions become a standard consideration in BIT formulations, especially with nations of comparable economic stature. This direction not only promises to enhance the health outcomes of BITs but also opens up a pathway for more nuanced future studies on the interplay between economic ties and public health objectives within IIAs. Furthermore, by building upon the efficacy of our machine learning approach, there is potential to extend this methodology to other domains of global governance and international relations, such as trade agreements, climate accords, and environmental treaties. This could unveil patterns and strategic alignments vital for crafting more cohesive and effective global policies in these areas, setting a clear agenda for future research directions.

Moreover, acknowledging additional factors such as per capita public health spending, political ties, political system designs, and, importantly, the frequency of a country being a respondent in Investor State Dispute Settlement (ISDS) cases related to public health, could provide a more comprehensive understanding of the motivations and outcomes associated with the inclusion of health safeguards in BITs. These aspects suggest a complex interplay of economic, political, and health-related considerations that could further elucidate the conditions under which health safeguards are integrated into international investment agreements, highlighting an area for future research to explore [36].

## Conclusion

This study introduces a novel method that combines country-level measures and network features with machine learning techniques to predict future BITs that include health provisions. We analysed BITs from an electronic database. Subsequently, countries were represented as nodes, with edges indicating BIT connections. Centrality metrics highlighted key nodes, and link prediction estimated future BIT ties, considering country-level economic indicators, such as GDP and population. The BIT network consists of 167 countries with 534 connections. On average, each country has BITs with six other countries. For link prediction, we utilised three tree-based models. XGBoost outperformed the others with an accuracy of 64.02%. The Common Neighbour centrality feature and the GDP Ratio were significant predictors for understanding BIT with health inclusion formations. Furthermore, analysing BIT networks across three periods and various types reveals a slight shift in feature importance. This shift reflects adjustments in global economic priorities, potentially impacting health protection provisions in BITs.

Despite the strengths of our approach, there are several limitations to consider. Our models rely heavily on the availability and accuracy of economic and network data, which may vary across different countries and time periods. While tree-based models offer interpretability, they may not capture the full complexity of international treaty negotiations compared to more advanced deep learning models. Additionally, our analysis focuses primarily on structural and economic factors, potentially overlooking other critical aspects such as political dynamics and public health priorities. Future research should explore the integration of more diverse data sources, including political and public health indicators, to provide a more holistic view of the factors influencing BIT formulations. There is also potential to apply state-of-the-art deep learning models, such as GNNs and large language models, to capture more intricate patterns and relationships within the data, while addressing interpretability challenges to ensure practical applicability for policymakers.

In summary, our research underscores the importance of integrating health safeguards into BITs. We examine the evolving dynamics of treaty formation and provide insights into the prospective frameworks of BITs and their potential implications for public health by employing ML techniques. Additionally, our dataset suggests that factors such as capital account balance and unemployment rates may influence the incorporation or likelihood of adopting health safeguards in BITs. These indicators often reflect a country’s economic stability and social priorities, which could indirectly shape negotiation dynamics. Our findings highlight the need for further exploration of how economic status and other factors might confer different types of negotiating power and leverage within international investment agreements. This analysis could enhance our understanding of a nation’s negotiation leverage and its commitment to public health measures within these frameworks.

## Data Availability

The data used in this study are publicly available (https://edit.wti.org/ document/investment-treaty/search).
